# Cinnamon in Anticancer Armamentarium: A Molecular Approach

**DOI:** 10.1155/2018/8978731

**Published:** 2018-03-29

**Authors:** Anindita Dutta, Anindita Chakraborty

**Affiliations:** UGC-DAE Consortium for Scientific Research, Kolkata Centre, Sector-III, Block-LB-8, Salt Lake, Kolkata, West Bengal 700106, India

## Abstract

In recent years, natural bioactive components draw a major attention for their potent anticarcinogenic activity. Cinnamon, one of the traditional spices, most frequently used in almost every household of tropical countries has got enormous efficacy to combat cancer. Cinnamon as a whole and/or its active components exhibited significant antineoplastic activity in different types of cancer. This review has been carried out to elicit the molecular mechanisms of action of cinnamon and its components on oncogenic regulators and related pathways. Web sites of Google Scholar, Medline, and PubMed were searched for articles written in English and published in peer-reviewed journals from 2003 to 2017. The anticarcinogenic potential of cinnamon varies with the type of cancer and also depends on the administered active compound individually or in combination with some chemicals or even extract of cinnamon as a whole. Some of its active components exert chemosensitization of well-known anticancer drugs. These outstanding properties of this spice necessitate its incorporation in both pharmaceuticals and nutraceuticals to explore possibilities of formulation of novel drug from this spice for treatment and prevention strategy of cancer.

## 1. Introduction

**Figure 1:**
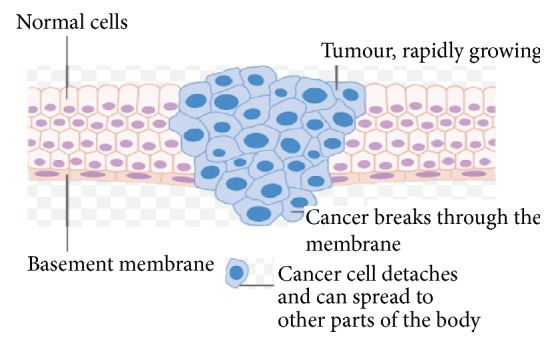
Schematic presentation of the basic properties of cancer cells (adapted from Cancer Research UK).

Despite the invention of numerous strategies to treat cancer, a rising burden of cancer is getting imposed on the world as indicated by the estimation of International Agency for Research on Cancer (IARC) in 2012. According to the report, in 2012, while the number of deaths in cancer accounts to 8.2 million, 14.1 million new cases had been recorded worldwide as compared to 12.7 million new cases in 2008, with 32.6 million cases of cancers in individuals aged ≥15 years (five-year prevalence). A substantive rise in new cancer cases as indicated by Globocan 2012 is estimated to 19.3 million by 2025 [1]. The constantly increasing incidence of cancer is making it an imperative matter of concern among the investigators of medicinal research. The principle types of cancer treatments are surgery, chemotherapy, radiotherapy, targeted therapy, immunotherapy, and some other techniques like stem cell transplantation. All of the established cancer treatments have some limitations and the principle obstacles with most frequently applied cancer therapy, that is, chemotherapy and radiotherapy, are recurrence and vast number of side effects, respectively. The basic difference between normal and cancer cells with regard to their attachment to the basement membrane has been shown in Figure 1.

Thus, in recent times, major attention is being devoted to discover additional novel strategies to combat cancer through natural dietary components. In this regard, the delineation of anticancer potential of phytochemicals is one of the supreme domains of current day research. Cinnamon, one of the most frequently used spices from ancient times, is being extensively investigated for its health promoting potentials, and among them its anticancer efficacy is at the prime focus.

**Figure 2:**
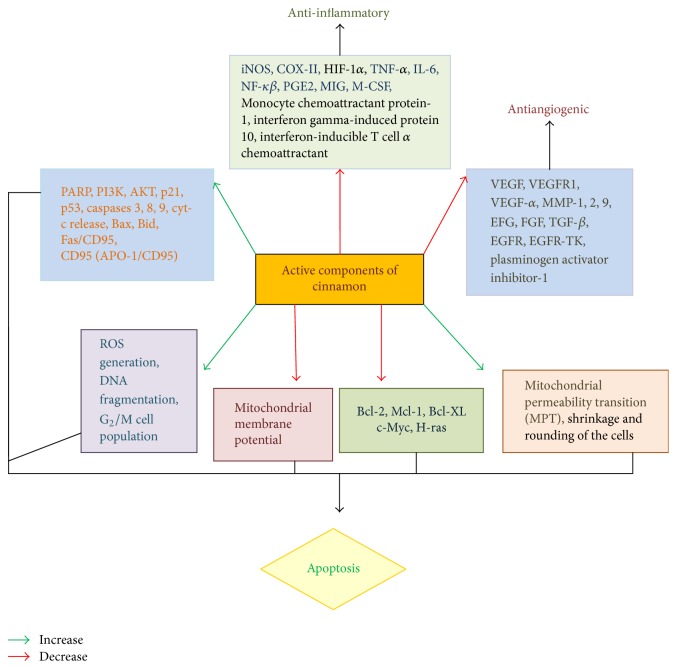
Action of active components of cinnamon on cancer cell growth and differentiation/apoptosis, necrosis, and senescence.

**Figure 3:**
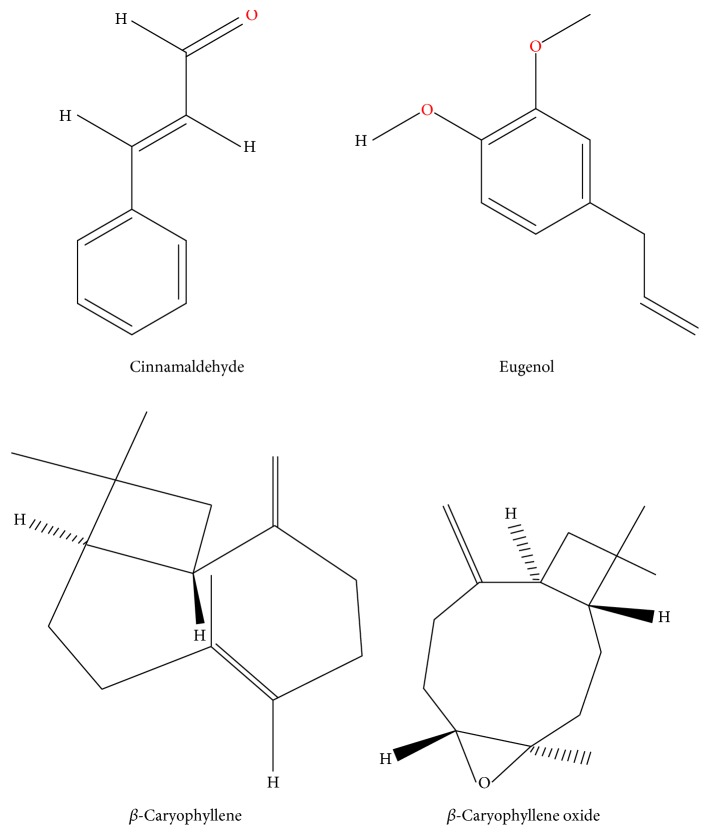
Chemical structures of different active compounds of cinnamon (adapted from PubChem Open Chemistry Database, NCBI).

Of late elucidation of the intricate mechanisms involved in the molecular aspect of interaction of cinnamon with development of malignancy has become an important area of exploration where it is felt that probing into the multifarious process, through which cinnamon brings about its anticarcinogenic efficacy, is immensely required for its implementation in the field of anticancer drug development. This review is an attempt to portray the potential of cinnamon as a whole and its active components in modulating neoplastic processes through influencing divergent biochemical pathways as reported through several in vivo and in vitro documentations (see Figure 2). The molecular mechanisms of action of cinnamon and its components as discussed would foment further initiative to include cinnamon in drug development research in the near future.  Figure 3 shows the chemical structures of some of the major active compounds of cinnamon.

## 2. Interaction with Cancer Cell Survival

The most apparent outcome of any potential anticarcinogenic compound is profound alterations in the viability of cancer cells. Several studies have shown strong antiproliferative potential of cinnamon against different cancer cells. Significant antiproliferative action of cinnamon extract has been observed in three different hematologic tumor cell lines Jurkat, Wurzburg, and U937 [2]. Although each of the cell types shows a dose-dependent reduction in percentage of cells when treated with a dose range of 0.05 to 0.2 mg/mL of cinnamon extract (CE) for 24 h, Wurzburg cells showed highest sensitivity towards CE. This establishes cell-specific differential potential of cinnamon in controlling cell proliferation. A cervical cancer cell line SiHa also showed antiproliferative action of cinnamon extract when treated with different concentrations of CE (10, 20, 40, and 80 *μ*g/ml) for three different time durations 24, 48, and 72 h [3]. This group observed a dose-dependent decrease in the growth kinetics with a twofold decrease at a concentration around 80 *μ*g/ml of CE treatment in comparison to the untreated SiHa cells. The antiproliferative potential of CE-c was further complemented through clonogenic assay.

Besides the extract of cinnamon, in recent times, different active compounds of cinnamon have also been reported to enhance cytotoxicity of various cancer cell types. Treatment with a concentration range of 10 *μ*M to 1 mM of eugenol for 24 h declined the proliferation rate of HeLa cells. While 500 *μ*M of eugenol showed no cytotoxicity on normal lymphocytes, the same concentration could induce 50% inhibition of growth of HeLa cells [4]. Cytotoxic effect of eugenol has also been observed against colon cancer cells HCT-15 and HT-29 where HCT-15 cells were found to be more sensitive than HT29 cells towards eugenol [5]. Eugenol exhibited remarkable antiproliferative efficiency against promyelocytic leukemia cell, HL-60, with an IC50 at a concentration of 23.7 mM [6]. Application of eugenol in combination with gemcitabine (anticancer drug) has significant chemosensitizing effect. A combination of gemcitabine (15 mM) and eugenol (150 *μ*M; sublethal dose) significantly amplifies the cytotoxic efficiency of both of these compounds towards HeLa cells (47% viable cells), compared with antiproliferative activity of either of the compounds applied individually (73% and 84% viable cells, respectively, for gemcitabine and eugenol), thus reflecting the possibility of using a lower dose [4]. Sensitizing effect of eugenol has also been observed in human prostate cancer cells where eugenol in combination with 2-methoxyestradiol (2ME-2) synergistically enhances its cytotoxicity against androgen independent PC-3 cell line compared to either of their individual effects. IC50 of both 2-ME2 and eugenol when applied alone was 1 *μ*M and 82 *μ*g/ml, respectively; however, the combination (0.5 *μ*M 2-ME2 + 41 *μ*g/ml eugenol) exhibited 50% growth inhibition. This combination further significantly suppressed anchorage independent growth of PC-3 cells in spite of no significant effect reported in case of individual application of either 2-ME2 or eugenol. Thus both anchorage dependent and independent growth of prostate cancer cells can be effectively decreased by this combination [7].

Cinnamaldehyde exhibited potent antiproliferative effect on a liver cancer cell line, HepG2, in a dose- and time-dependent pattern where a concentration of 30 *μ*M of cinnamaldehyde inhibited approximately 71% of cell proliferation [8]. Similarly PLC/PRF/5 cells (another liver cancer cell line), treated with the same compound, showed decreased cell viability in a time- (6, 12, and 24 h) and concentration-dependent (0.1, 0.5, 1, and 5 *μ*M) manner [9]. Another in vitro study reported marked cytotoxic effect of cinnamaldehyde on HL-60 cells with an IC50 value of 30.7 mM [10].

*β*-Caryophyllene (CPO), a major component of cinnamon, has also been reported to modulate cancer cell survival pathways. In prostate (PC3) and breast cancer (MCF-7) cells, administration of *β*-caryophyllene (CPO) for 6 h significantly hindered the constitutive activation of PI3K and serine/threonine protein kinase AKT in a dose-dependent pattern (10, 30, and 50 *μ*M) which directly decreases the rate of cell proliferation [11].

Besides the antiproliferative activity of cinnamon, distinct alterations in the morphology of the cancer cells have also been observed with application of cinnamon, either as extract or as individual active constituent compound(s). In a mouse melanoma model, incorporation of cinnamon extract, orally or via intratumoral injection, notably decreased the growth of tumor after 22 days of treatment as detected by measuring its weight. It significantly inhibited the incidence of metastasis by markedly decreasing weight and size of the draining spleens and lymph nodes, in comparison with the control [12]. Pretreatment with eugenol in DMBA croton oil induced skin carcinogenic mice model significantly suppressed the neoplastic morphological changes, restricting it at the premalignant stage. The size of the papillomas has been observed to be three times smaller in the eugenol treated group as compared to that of the untreated tumor-bearing mice. It markedly restricted the process of carcinogenesis at the dysplastic stage with only thickening of the epithelial layer or acanthosis, whereas in the control group attributes indicative of advanced squamous cell carcinoma include hyperkeratosis, keratin pearl development, and acanthosis [13]. A combination treatment of prostate cancer PC-3 cells with eugenol (41 *μ*g/ml) and 2-methoxyestradiol (0.5 *μ*M) for 24 h resulted in remarkable alterations in the morphology of the cells, compared to either of their individual effects. The observed morphological changes include shrinkage and rounding of the cells and abrogation from the surrounding cells [7].

## 3. Effect on Growth Factors

In cancer therapy, modulation of expression of growth factors is one of the most crucial targets. In mouse melanoma cell lines (B16F10 and Clone M3), CE markedly hindered the transcription and translation level of different growth factors (EFG, FGF, VEGF-*α*, and TGF-*β*) in a concentration-dependent (0.3 or 0.5 mg/ml) fashion, whereas no such effects have been observed when the same concentration of CE was applied in normal cells [12]. Similarly, essential oil of cinnamon (EOC) applied to xenograft model of Hep-2 cells (laryngeal squamous cell carcinoma) inhibited the EGFR-TK (epidermal growth factor receptor-tyrosine kinase) activity (89% inhibition noted) resulting in 43.5% decrease in the tumor burden [14]. In preinflamed human dermal fibroblast system, cinnamon bark essential oil (CBEO) from* Cinnamomum zeylanicum* exhibited similar effects by notably downregulating the expression levels of epidermal growth factor receptor (EGFR), plasminogen activator inhibitor-1, and matrix metalloproteinase 1 [15].

## 4. Cinnamon on Cell Cycle

Cinnamon extract (CE) has been found to alter the cell cycle phases in three different hematologic tumor cell lines, Jurkat, Wurzburg, and U937 cells in a concentration-dependent manner. However, the effect of CE is not same in all the phases of cell cycle in the three different cell types. While in the G0/G1 phase, all the three cell lines showed >70% loss of cell population with CE treatment, in the S phase, maximum effect was seen in case of Wurzburg cells (>4-fold decrease in cell population with 0.2 mg/mL CE), and no significant alterations have been observed for Jurkat and U937 cells. Arrest at the G2/M phase was also maximum in case of Wurzburg cells. There was an approximate 10-fold increase in the number of Wurzburg cells as compared to only ~3.5-fold increase in case of both Jurkat and U937 cells [2].

Active compounds of cinnamon have also been observed to impart significant changes in the cell cycle of cancer cells. Treatment with eugenol significantly enhanced cell cycle arrest in colon cancer cell lines, namely, HCT-15 (treated with 300 *μ*M Eugenol) and HT-29 (treated with 500 *μ*M Eugenol) cells in a time-dependent manner observed up to 48 h [5]. In prostate cancer cell PC3, synergistic action of eugenol (41 *μ*g/mL) in combination with 2-methoxyestradiol (0.5 *μ*M) for 24 h could greatly enhance the rate of cell cycle arrest at the G2/M phase (4.6-fold increase in arrest over that of the PC3 cells treated individually with eugenol or 2ME-2) [7]. Cinnamaldehyde remarkably induced cell death through alteration in the distributions of cells in the different phases of cell cycle in case of PLC/PRF/5 hepatoma cell line. 1 *μ*M cinnamaldehyde increased the sub-G1 population from 13.80% to 66.70% (at 6 h to 24 h, resp.) followed by increase in S phase cells and decrease in both G0/G1 and G2/M phase [9].

## 5. Role of Cinnamon on Apoptosis

Apoptosis or programmed cell death, a complex interplay among numerous functional molecules and pathways, is one of the most prime factors to be targeted by many anticancer treatment strategies. Extract of cinnamon as a whole and its different active compounds exhibit significant alteration in the rate of apoptosis in different types of cancer. Effect of cinnamon has been worked out on apoptotic pathways where it is established that cinnamon extract stimulates caspase-3 activity in in vivo melanoma model and PMA + ionomycin induced B16F10 cells. However, although the procaspase-3 (inactive) level remained unaltered in this treatment, expressions of NF*κ*B and AP1 and transcription and translation levels of their target genes, Bcl-2, BcL-xL, and survivin, have been observed to be highly decreased [16].

Eugenol, one of the most important active compounds of cinnamon, is found to induce apoptosis in promyelocytic leukemia cell, HL-60, in a time- and dose-dependent pattern through a marked increase in the rate of DNA fragmentation and loss of mitochondrial potential via ROS generation [6]. Increased ROS generation by eugenol has also been reported in colon cancer HCT-15 and HT-29 cells [5]. Treatment of HL-60 cells with 40 *μ*M eugenol for 4 h resulted in significant fragmentation of DNA through cleavage at internucleosomal linker regions, forming an apparent DNA ladder. In addition to DNA fragmentation, proteolytic cleavage of caspase-3 and caspase-9 from their respective proforms also accounts for apoptosis induction by eugenol. However, at this concentration of eugenol treatment, although there occurs significant depletion in the level of bcl-2 protein together with enhanced cytochrome c release, no notable effect has been noticed in the cleavage of procaspase-8 [6]. An in vivo work showing 1.5-fold increase in the apoptotic index with eugenol pretreatment in DMBA/TPA induced murine skin carcinogenesis via significant upregulation in the expression of p53 p21^WAF1^ proves the efficacy of this active compound of cinnamon in modulating apoptosis [17]. Potential of eugenol has also been shown in modulation of Bax: Bcl2 ratio in addition to upregulation of caspase-3 activity in chemically induced murine skin carcinogenesis [13]. In HeLa cells too, eugenol exhibited a concentration-dependent upregulation in the level of caspase-3 activity. Moreover, eugenol exerted a significant chemosensitizing effect when applied in combination with a well-known chemotherapeutic drug, gemcitabine (150 *μ*M eugenol with 15 mM and 25 mM gemcitabine), by synergistically amplifying the activity of caspase-3, in comparison to their individual effects [4]. Another research group also showed better potential of eugenol to induce apoptosis when administered in combination with 2-methoxyestradiol, in prostate cancer PC-3 cells through marked downregulation of Bcl-2 protein and upregulation in the expression of proapoptotic protein Bax and also influencing loss in mitochondrial membrane potential [7].

**Figure 4:**
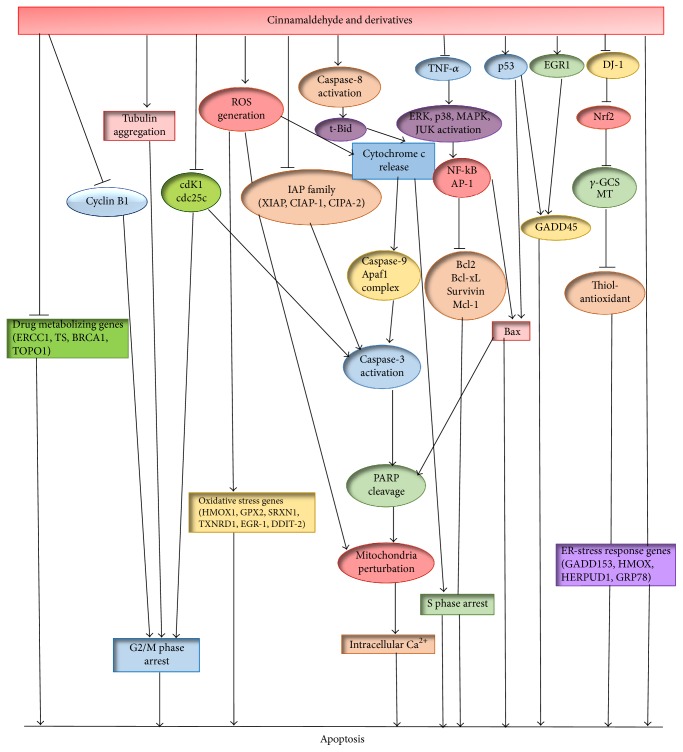
Schematic diagram of the mechanisms of action by which cinnamaldehyde and its derivatives induce apoptosis in cancer cells (adapted from* Phytother. Res.* 30: 754–767; DOI: 10.1002/ptr.5592, 2016).

Similar to eugenol, cinnamaldehyde (40 *μ*M) has been observed to induce apoptosis in HL-60 cells through internucleosomal fragmentation of DNA together with chromatin condensation and degradation of nuclei. Significant upregulation of the proteolytic cleavage of procaspase-9 and procaspase-3 followed by enhanced activity of caspase-3 and caspase-9, loss in mitochondrial transmembrane potential, and the release of cytochrome c into the cytosol has also been noted by this group [10]. At 30 *μ*M concentration, cinnamaldehyde exhibited marked anticarcinogenic effect on liver cancer cell, HepG2, by decreasing the antiapoptotic protein Bcl-XL level and increasing the level of proapoptotic protein Bax together with p53 in a time-dependent manner. Complete suppression of Bcl-XL expression was observed after 24 h of cinnamaldehyde treatment. Cinnamaldehyde at this concentration also upregulates CD95 (APO-1/CD95) expression and poly (ADP-ribose) polymerase (PARP) cleavage [8]. Much lower concentration of cinnamaldehyde (1 *μ*M) has been reported to induce apoptosis via upregulation of caspase-8 activity, Bax, and Bid together with marked decrease in the expression of antiapoptotic proteins (Bcl-2, Mcl-1) in hepatoma cell line PLC/PRF/5 cells in a time-dependent pattern (6, 12, and 24 h) [9]. An isomer of cinnamaldehyde, trans-cinnamaldehyde, increased the rate of apoptosis in myelogenous leukemia cell line K562 cells, mediated through the loss of mitochondrial transmembrane potential, and involved enhanced expression of Fas/CD95 [18].  Figure 4 schematically presents the mechanisms of action by which cinnamaldehyde and its derivatives exert their anticarcinogenic potential [19].

*β*-Caryophyllene oxide (CPO) markedly raised both the early and late apoptotic population of prostate cancer PC-3 cells by significantly enhancing the DNA strand breaks. Concomitant to induce apoptosis, it has also been shown to induce loss of mitochondrial membrane potential resulting in mitochondrial dysfunction in prostate cancer cells [11].

## 6. Effect on Immune Response and Inflammation

Immunotherapy has become an important strategy for combating cancer as immunity interrelated with inflammatory processes has an important role in cancer development. Application of natural components in boosting the immune system has recently been given prime focus. CD8+T cell or cytotoxic T lymphocyte or killer T cell acts as a key factor in limiting the process of carcinogenesis. Cinnamon extract administration, either orally (OA) or via intratumoral injection (IT), significantly reduced the rate of tumor cell proliferation in vivo through greatly accelerating the cytolytic activity of CD8+T cells. An increased expression of cytolytic factors, IFNc and TNF-a, has been observed in CE treated cells as compared to the control. The treatment also resulted in marked upregulation in the level of granzymes (granzymes B and C) and perforin protein concomitant to induce programmed cell death [12]. IL-1*β* is one of the contributing inflammatory factors in carcinogenesis. In HeLa cells, eugenol administration completely subdued the expression IL-1*β* as compared to the respective control [4]. Another study reported that eugenol pretreatment hinders in vivo tumorigenesis by significantly inhibiting 12-O-tetradecanoylphorbol-13-acetate (TPA) induced upregulation in the expression levels of proinflammatory cytokines including interleukin 6 (IL-6), tumor necrosis factor alpha (TNF-*α*), and prostaglandin E2 (PGE 2). The expressions of these cytokines remained almost the same as that of the untreated and control level, when pretreated with eugenol for 30 min before getting exposed to TPA [17]. Essential oil of cinnamon bark (CBEO) from the species* Cinnamomum zeylanicum* depleted expressions of certain inflammatory cytokines, namely, interferon gamma-induced protein 10, interferon-inducible T cell alpha chemoattractant, monocyte chemoattractant protein-1, and monokine induced by gamma interferon (MIG) in human dermal fibroblast system. A marked decrease in the level of an immunomodulatory protein, macrophage colony-stimulating factor (M-CSF), has also been observed with CBEO application [15].

## 7. Impact on Angiogenesis and Oncogenes

Angiogenesis is an indispensable factor in progression of cancer as it is an essential process for the metastasis of cancer cells. In this process, hypoxia inducible factor 1 alpha (HIF-1*α*) and cyclooxygenase 2 (COX-2) play pivotal roles. In B16F10 and Clone M3 mouse melanoma cells, administration of cinnamon extract for 24 h markedly downregulated the expression level of both Cox-2 and HIF-1*α* in a concentration-dependent (0.1, 0.2, 0.3, 0.4, and 0.5 mg/ml) pattern, with complete restriction of expression of Cox-2 at 0.5 mg/ml of CE [12]. Cinnamon extract exhibited similar effects in vivo (mouse melanoma model) treated either orally (OA) or via intratumoral injection (IT) for 30 days. The oral administration of the extract showed more potential in downregulating both transcription and translation level of HIF-1 *α* and Cox-2 than the intratumoral injection (IT). The transcription level of Cox-2 decreased 40% in the IT group whereas a 90% reduction has been reported in the OA group [12]. Similar to CE, eugenol treatment remarkably decreased expression of COX-2 in HeLa cells [4]. Eugenol pretreatment significantly restricted the transcription level of COX-2 and iNOS in TPA induced mouse skin cancer model [17]. Pretreatment with eugenol has also been observed to impede DMBA croton oil inducing the skin carcinogenesis in mice by greatly reducing the transcription and translation level of two oncogenes, c-Myc and H-ras. In case of c-Myc, a 40% decrease in the expression and for H-ras 28% downregulation have been reported as compared with control [13].

## 8. Conclusion

Cinnamon has long been used in the culinary field for its distinct aroma and flavour. Although its application in the medicinal purposes has been mentioned in ayurveda and of late its pharmacological potency is being extensively investigated, significance of this spice in cancer has not yet been explored to the fullest at the molecular level. The present review offers a compilation of information which reflects a profound anticarcinogenic efficacy of cinnamon, brought about by modifying multiple oncogenic targets. Data gathered from literature survey suggest that application of cinnamon or its active compounds effectively hinders the process of cancer development at different stages from initiation to metastasis, preventing its onset to delaying its progress. This review article presents a molecular approach unravelling the interaction of cinnamon with various pathways and functional biomolecules that suffer perturbations in the process of cancer development. The work envisages future possibility of incorporation of this age-old spice and/or its active components in pharmaceutical formulations meant for chemoprevention as well as chemosensitization. This might provide space to initiate further analytical investigations and introduce cinnamon for development of novel anticancer drug.
